# Global incidence and risk factors for injury-related bloodstream infections: a scoping review

**DOI:** 10.1093/epirev/mxaf015

**Published:** 2025-10-24

**Authors:** Binuri Perera, Ashleigh Earnshaw, Kevin Laupland, Samantha Borg, Kirsten Vallmuur, Felicity Edwards, Susanna Cramb

**Affiliations:** The Australian Centre for Health Services Innovation, School of Public Health and Social Work, Faculty of Health, Queensland University of Technology, Brisbane, QLD, Australia; Jamieson Trauma Institute, Metro North Health, Brisbane, QLD, Australia; The Australian Centre for Health Services Innovation, School of Public Health and Social Work, Faculty of Health, Queensland University of Technology, Brisbane, QLD, Australia; Department of Intensive Care Services, Royal Brisbane and Women’s Hospital, Brisbane, QLD, Australia; School of Medicine, Faculty of Health, Queensland University of Technology, Brisbane, QLD, Australia; The Australian Centre for Health Services Innovation, School of Public Health and Social Work, Faculty of Health, Queensland University of Technology, Brisbane, QLD, Australia; Jamieson Trauma Institute, Metro North Health, Brisbane, QLD, Australia; The Australian Centre for Health Services Innovation, School of Public Health and Social Work, Faculty of Health, Queensland University of Technology, Brisbane, QLD, Australia; Jamieson Trauma Institute, Metro North Health, Brisbane, QLD, Australia; Department of Intensive Care Services, Royal Brisbane and Women’s Hospital, Brisbane, QLD, Australia; School of Medicine, Faculty of Health, Queensland University of Technology, Brisbane, QLD, Australia; The Australian Centre for Health Services Innovation, School of Public Health and Social Work, Faculty of Health, Queensland University of Technology, Brisbane, QLD, Australia; Jamieson Trauma Institute, Metro North Health, Brisbane, QLD, Australia

**Keywords:** incidence, risk factors, injuries, bloodstream infection, review, epidemiology

## Abstract

Bloodstream infection (BSI) can be a serious complication among injured patients. Understanding the burden of injury-related BSI is important for early detection and implementing appropriate treatments to improve patient outcomes. Incidence rates and risk factors are important measures that help provide insights into the burden of injury-related BSIs and early diagnosis of patients. In this review, the aim was to comprehensively summarize incidence rates and risk factors for injury-related BSIs from scientific literature. Four electronic databases (PubMed, CINAHL [via EBSCOhost], Embase, and Web of Science) were searched. There were no limitations on the language. Studies reporting the incidence rates or risk factors associated with incidence or adverse outcomes from injury-related BSIs were included. Database searches returned 9830 articles, of which 48 were included. Incidence rates of injury-related BSIs ranged from 0.71 to 27.4 episodes per 1000 patient-days. A total of 237 potential factors associated with the development and/or outcomes of injury-related BSIs were identified and classified into 8 broad categories: demographics, prognostic scores, burn extent, clinical and patient health factors, biomarkers, resource utilization and treatments, pathogens and injuries, and mechanisms. Older age, male sex, higher injury severity score, longer length of stay, greater total body surface burn area, and inhalation injuries were the most frequently reported risk factors. This review identified a large variation in reported incidence rates but no population-based studies. Many factors have been associated with injury-related BSIs; however, the direction of association and effect sizes vary across the studies, which can be attributed to the differences in study design.

## Introduction

Bloodstream infections (BSIs) cause a significant burden on patients and health care systems worldwide.^1‑3^ Bloodstream infections occur when viable pathogenic microorganisms enter the bloodstream; a positive blood culture considered the diagnostic requisite. Depending on the type of microorganism present in the bloodstream, BSIs can be associated with bacteremia or fungaemia, indicating the presence of bacteria or fungi in the bloodstream, respectively. Bloodstream infections may progress to sepsis or septic shock, life-threatening conditions characterized by organ dysfunction caused by a dysregulated host response to infection.^4^ Early identification of the potential factors for these conditions is crucial to prevent adverse events, including death, and implement targeted interventions to improve patient outcomes.^5^

Among injured patients, BSIs can result in severe complications, including prolonged hospital stays, increased use of antibiotics, increased resource utilization,^6^ and increased risk of in-hospital death.^5‑12^ Furthermore, injured patients have a higher risk of acquiring BSIs, given that there can be damage to the skin, a weakened immune system, and the use of invasive devices (eg, catheters, intravenous ports).^13^^,^^14^

Despite the importance of understanding risk factors for injury-related BSIs, our current overall knowledge of the burden of injury-related BSIs is limited. We identified no previous literature reviews on the incidence and risk factors of injury-related BSIs. Although studies have examined the incidence and risk factors of BSIs associated with injured patients in specific clinical settings,^5^^,^^15^ for specific pathogens,^16^^,^^17^ age groups,^5^^,^^6^^,^^15^ and injury types,^9^^,^^17^^,^^18^ no comprehensive study, to our knowledge, has been made of all patients with injury-related BSIs to date. In this scoping review, we aim to summarize existing evidence on the incidence and risk factors for developing or adverse outcomes from an injury-related BSI.

## Methods

This review was conducted in accordance with an a priori protocol registered with the Open Science Framework (https://doi.org/10.17605/OSF.IO/EKYNT), dated September 3, 2024, and reported in compliance with the JBI methodology for scoping reviews.^19^ We explored and mapped the existing literature examining the occurrence and determinants of the development and outcomes of injury-related BSIs among all age groups.

The following research questions were set to achieve our aim:

What is the overall incidence of injury-related BSIs?
What are the risk factors associated with the incidence of injury-related BSIs?
What are the risk factors associated with adverse outcomes of injury-related BSIs?

### Inclusion criteria

#### Participants

Studies included human participants of any age who were admitted to hospital-based settings, including trauma centers, emergency departments, and intensive care units, due to traumatic injuries and who subsequently developed a BSI, as diagnosed through a positive blood culture. The number of patients with injury-related BSIs had to be at least 10, and studies focusing on autopsies and nontraumatic injuries (e.g., acute kidney injury, mucosal barrier injury, veterans with ambiguous spinal cord injuries) were excluded.

#### Concept

This review included studies that reported incidence rates or investigated risk factors of BSIs specifically related to traumatic injuries. Articles on sepsis, septicemia, and septic shock in injured patients were included if those articles reported the use of blood cultures for diagnosis. However, studies that investigated diagnosing BSIs or sepsis using biomarkers, the molecular epidemiology of microorganisms causing BSIs, preventive strategies, or quality improvement for injury-related BSIs were excluded.

#### Context

This review included articles published since January 1, 1993. Studies conducted in clinical settings, such as hospitals, trauma centers, emergency departments, and intensive care units, were considered with no country or language restrictions.

### Types of sources

All original research articles were considered, excluding case reports, review articles, editorials and abstract-only articles.

### Search strategy

The search strategy included terms related to BSI, injuries, incidence, and risk factors to describe the characteristics and outcomes (Table S1). The strategy was discussed with all authors and developed in consultation with a liaison librarian. The databases used were PubMed, Embase (not including Medline content), CINAHL (via EBSCOhost), and Web of Science. The study search using the developed search strategy was conducted on July 2, 2024.

### Source of evidence selection

**Figure 1:**
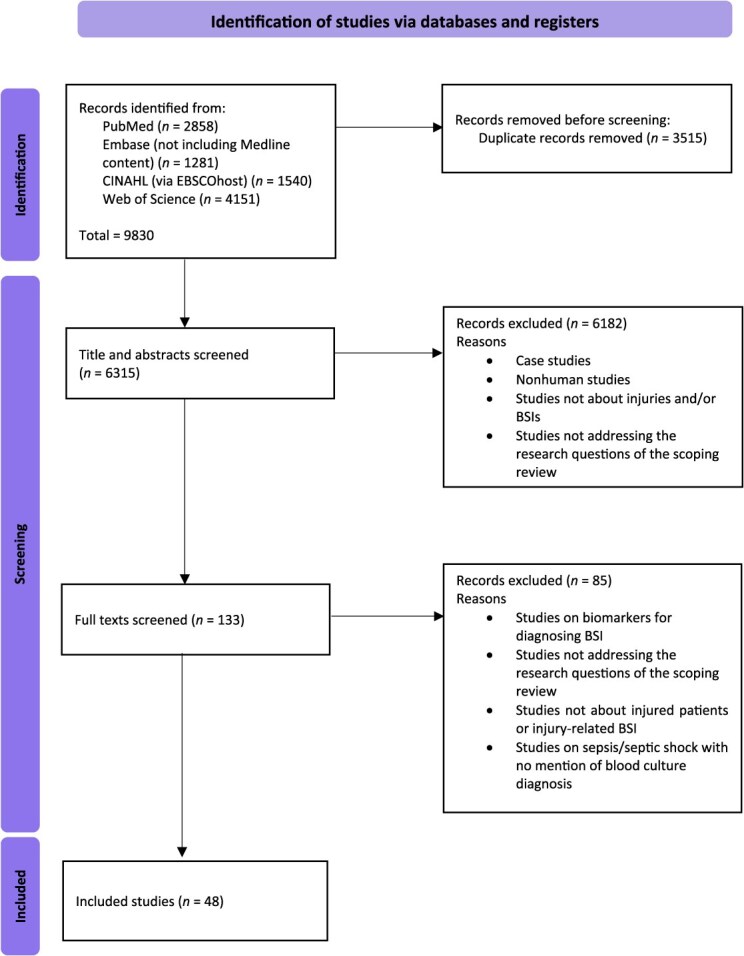
Preferred Reporting Items for Systematic Reviews and Meta-Analyses (PRISMA) diagram.

All potential studies identified from the databases were uploaded to EndNote 21^20^ and then into Rayyan^21^ to remove duplicates. Title and abstract screening of articles was independently conducted by 2 reviewers (B.P. and A.E.) in Rayyan, and discrepancies were resolved by a third reviewer (S.C.). Full-text screening was then completed independently against the same eligibility criteria by the same 2 reviewers. The search results and the study inclusion process are presented in a Preferred Reporting Items for Systematic Reviews and Meta-Analyses (PRISMA) flow diagram (Figure 1).

### Data extraction

Data were extracted from included articles, using a data extraction tool developed by the reviewers (Table S2). Data extraction was performed by 1 reviewer (B.P.) and 10% of the data extraction from all included studies was validated by 2 independent reviewers (B.P. and A.E.). The data extraction included specific details about the participants, concept, context, study methods, and key findings relevant to the review questions.

### Data analysis and presentation

The relevant studies identified were summarized, addressing the objectives and the research questions, with results presented in tables and figures. The rates of injury-related BSIs from all the included studies were calculated as the cumulative incidence (i.e., the proportion of people who developed a BSI compared with the total population of interest).

## Results

### Search results

The searches of the 4 electronic databases resulted in 9830 articles with PubMed (*n* = 2858), Embase (*n* = 1281), CINAHL (*n* = 1540), and Web of Science (*n* = 4151). After deduplicating, the number of citations was reduced to 6315, and 48 articles remained for data extraction after title and abstract screening and the application of inclusion-exclusion criteria (Figure 1).

#### Characteristics of the included studies

Of the 48 studies, 68.8% (*n* = 33) focused on patients with burn injuries^7‑12^^,^^16‑18^^,^^22‑45^ and the subsequent 15 focused on all injury types combined^5^^,^^6^^,^^13‑15^^,^^46‑55^; only 1 study^15^ of these did not include patients with burn injuries. Most of the included studies reported a specific BSI organism: 16 studies focused on bacteremia only and 7 had a major focus on candidemia. Two articles specifically discussed sepsis and septic shock. The remaining 23 studies were not restricted to a specific organism but could be classified based on the type of BSI: 11 articles covering all types of BSIs, 7 discussing catheter-related BSIs, 3 articles on patients with nosocomial BSIs, and an article each on persistent BSIs and all infections. Among the included studies focusing on patients with burn injuries, *Pseudomonas aeruginosa* was the most identified pathogen^9^^,^^26^^,^^29-31^^,^^43^ followed by *Acinetobacter baumannii.*^28^^,^^38^^,^^39^^,^^41^^,^^56^ The study periods of the studies reported on in the articles ranged from 90 days^39^ to 21 years.^33^^,^^34^ Time to BSI was reported in 17 studies and ranged from 5 to 41 days (Table S3).

Although most included articles focused either on mixed (adult and pediatric) or adult patients, 4 articles specifically discussed the incidence and risk factors of injury-related BSIs in pediatric populations.^18^^,^^35^^,^^43^^,^^45^ Among the studies that included mixed populations, the minimum age ranged from 3 years to 17 years (Table S3). Five articles reported only cohort-level characteristics and did not specify characteristics for those with and without a BSI.^14^^,^^28^^,^^37^^,^^52^^,^^53^ The median age range of hospitalized patients with an injury-related BSI was 35-60 years across the reviewed articles. In all but 1 article,^36^ male patients were reported to have more injury-related BSIs than females.

The 48 included studies consisted of cohort studies (*n* = 37), case-control studies (*n* = 5), chart reviews (*n* = 3), and surveillance/lab-based studies (*n* = 3) (Table S1). Most studies were conducted in single centers (*n* = 41; 85.4%), with few multicenter studies (*n* = 7; 14.6%). Of the 7 multicenter studies, 2 reported data from more than 700 centers.^53^^,^^55^ Sample size varied from 58 (patients with burn injuries from a dust fire incident in China)^38^ to 175538 (trauma patients with central-line placement in a multicenter study).^55^ Studies represented 22 countries (Table S3), with most based in high-income countries (*n* = 40; 81.2%), 6 from upper-middle-income countries, and 2 from lower-middle-income countries, as defined by the World Bank^57^ (Table S3).

#### Incidence rates

**Figure 2:**
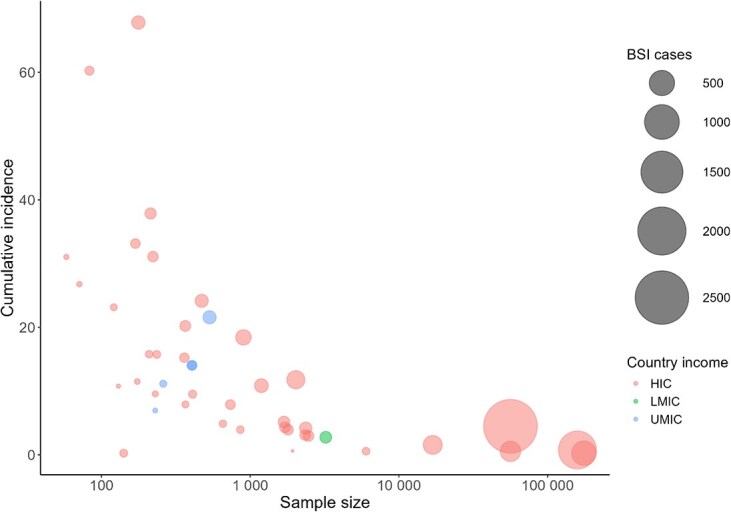
Variation of cumulative incidence, with the sample size. Abbreviations: HIC, high-income countries; LMIC, lower-middle-income countries; UMIC, upper-middle-income countries.

Fifteen studies (31.3%) reported the incidence rate^5^^,^^7^^,^^15^^,^^16^^,^^26^^,^^28^^,^^29^^,^^31^^,^^39^^,^^43^^,^^48‑51^^,^^56^ as the total number of infected patients or episodes per patient-days, hospital-days, or central line–days. The reported incidence rate for BSI among injured patients ranged from 0.71 episodes per 1000 patient-days (at an Indian level-1 trauma center during April 2008 to December 2009)^51^ to 27.4 episodes per 1000 patient-days (patients with severe burn injuries in multicenter tertiary hospitals in China during 2014)^39^ (Table S4). The cumulative incidence could not be calculated in 6 instances because the sample size was not reported^16^^,^^23^^,^^42^^,^^45^^,^^47^; thus, the number of episodes were reported instead of the number of cases.^41^ Studies reported the number of cases and/or episodes with BSIs ranging from 14^11^ to 1214.^6^ The cumulative incidence for 45 studies in which the cumulative incidence could be determined ranged from 0.2% (among central line–associated BSIs)^55^ to 67.8% (among BSIs in patients with severe burns from an industrial disaster)^39^ (Figure 2). The pooled cumulative incidence across all studies was 1.47%, and the median cumulative incidence was 10.85% (Table S4).

#### Overview of risk factors

**Figure 3:**
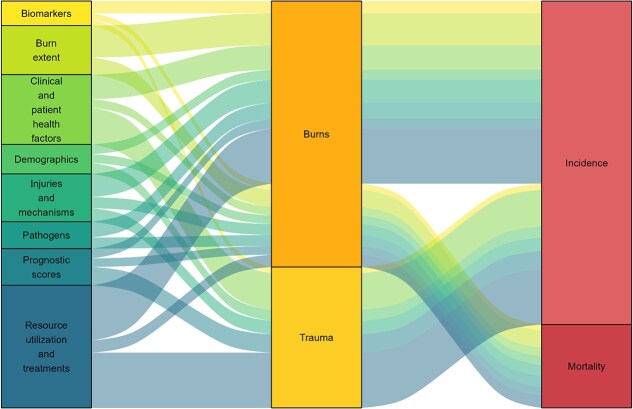
Sankey diagram showing the risk factors by type of trauma and outcome of interest.

Of the 48 included studies, 41 (85.4%) reported a total of 237 potential factors associated with injury-related BSIs (Table S5). Risk factors were assessed using bivariate analysis, logistic regression, or Cox regression. The effect sizes of outcomes were reported as odds ratios (ORs), relative risk ratios, hazard ratios, or regression coefficients. The 237 risk factors identified were grouped into 8 broader categories (Figure 3): clinical and patient health factors (*n* = 73; 31%); resource utilization and treatments (*n* = 61; 26%); injuries and mechanisms (*n* = 33; 14%); pathogens (*n* = 25; 11%); biomarkers (*n* = 18; 8%); prognostic scores (*n* = 9; 5%); burn extent (*n* = 9; 4%); and demographics (*n* = 4; 1%). Clinical and patient health factors were the variables most accounted for; however, there was considerable heterogeneity among individual variable types. Thirty-seven studies (90%) reported potential risk factors for either developing BSIs or experiencing adverse outcomes; the remaining 5 studies (10%) reported both.^10^^,^^12^^,^^18^^,^^37^ Twelve of the 40 studies (30%) included trauma patients combined and only reported risks for the development of BSI infections. The remaining 29 studies (73%) exclusively looked at cohorts of patients with burn injuries: 19 (66%) examined risk factors for developing BSIs in patients with burn injuries, and the remaining 15 (52%) reported risk factors relating to adverse outcomes, including death and longer hospital stays (including the 5 studies reporting potential factors for both developing and adverse outcomes). The risk factors identified are reported in the following paragraphs as 1) factors associated with developing injury-related BSIs and 2) factors associated with adverse outcomes from injury-related BSIs.

#### Risk factors for developing BSIs

Thirty-two studies reported potential factors associated with developing BSIs in injured patients. The most frequently included variables were age, injury severity score (ISS), total body surface area, length of stay, use of a ventilator, and inhalation injuries. Among these variables, where age and ISS were included, both variables were associated with increasing risk across studies. However, there was significant heterogeneity among other considered variables, including some with very large effect sizes, that only demonstrated statistical significance in a single study. Specifically, the white blood cell to platelet ratio (OR = 687.1)^44^; prior colonization with *A. baumannii* (OR = 26.2)^36^; having 2 or fewer debridements (hazard ratio = 26.4)^43^; and patients with a central venous catheter (OR = 22.1),^46^ gastrointestinal complication (OR = 20.37),^32^ or nongastrointestinal thromboembolic complication (OR = 17.3)^32^ had extremely large effect sizes, but only with respect to their specific study population (Table S5, Figures S1 and S2).

There was substantial heterogeneity in the variable selection and analyses among all studies that reported risks for trauma cohorts. The variables that were important across trauma cohorts for risk of BSIs were age, sex, ISS, length of stay and ventilator use. Specifically, BSIs were higher among male patients,^6^^,^^52^ with aging,^6^^,^^46^ increased ISS,^5^^,^^6^^,^^47^^,^^55^ and the use of ventilators.^6^^,^^48^^,^^52^ Length of stay was also often considered, but the direction and strength of findings were different.

Among the cohorts of patients with burn injuries, the variables that most consistently demonstrated associations with risk were age, sex, total burn surface area (TBSA), length of stay, inhalation injuries, and degree of burn. In contrast to patients with trauma, studies on burns identified increased risk among female patients^32^^,^^36^ and younger patients.^18^^,^^32^ Inhalation injuries were associated with an increased risk of BSIs among patients with burn injuries who had candidemia.^8^^,^^32^ Similar to the trauma cohort studies, there was mixed evidence for length of stay across studies.^17^^,^^18^

Total burn surface area was considered in most of the 29 burn studies. However, the classification of TBSA differed. It was unspecified in 14 studies,^8^^,^^9^^,^^11^^,^^17^^,^^18^^,^^24^^,^^26^^,^^27^^,^^31^^,^^32^^,^^35^^,^^40^^,^^42^^,^^56^ classified as ≥ 20% of TBSA in 3 studies,^12^^,^^37^^,^^43^ and considered in a single study for each of the following cut points: ≥ 30% of TBSA,^41^ ≥ 35% of TBSA,^34^ or > 50% TBSA.^36^ Greater TBSA was associated with increased risk across the studies, but the strength of the association varied as TBSA was unspecified (OR = 1.05)^17^; TBSA ≥ 30% (OR = 2.5)^41^; TBSA > 20% (hazard ratio = 11.06)^43^; and TBSA unspecified (OR = 53.7).^42^

Risk factors also differed by BSI type. For example, key risk factors uniquely identified for bacteremia included urinary tract infection and perineal burns,^12^ longer surgery time,^37^ hydrotherapy, and prior colonization with *A. baumannii.*^36^ Similar variable-specific effects were seen regarding other BSI types (Table S5). For studies focusing on catheter-related BSIs among patients with burn injuries, variables found to be important included catheter insertion sites,^56^ femoral placement and nonflame burns,^41^ and central-line duration.^26^^,^^56^ These differences are largely attributable to the variables being considered by a single study, again reinforcing the heterogeneity in modeling approaches and considered variables across studies. Similar findings are seen within the 4 pediatric-specific cohorts, in which there was evidence from a single study from specific variables, namely the degree of burn and debridement,^43^ and duration of mechanical ventilation for the development of candidemia.^18^

#### Risk factors associated with adverse outcomes for injury-related BSIs

Only 15 studies of patients with burn injuries examined factors associated with adverse outcomes after an injury-related BSI. Among these, there were similar patterns regarding variation in model choice and variable selection. Similar to the factors associated with developing BSIs, age, sex, TBSA, and inhalation injury data were reported in most of the studies (Table S5, Figures S3 and S4).

The factors that were specifically associated with increased risk of mortality in patients with burn injuries and bacteremia were increased age,^30^^,^^31^^,^^33^ higher ISS,^12^^,^^30^^,^^42^ higher sequential organ failure assessment score,^31^^,^^44^ a greater difference in the monocyte count from the first surgery to 3 days after the first surgery,^44^ hemofiltration,^33^^,^^34^ persistent bacteremia,^42^ recurrent bacteremia,^12^^,^^31^ bacteremia with *Klebsiella pneumoniae,*^30^ and perineal burns.^12^ Thrombocytopenia^23^ and gram-negative BSI^24^ were observed as independent risk factors for death in patients with burn injuries who had broader BSIs. Only 2 articles reported the risk factors affecting the mortality of patients with burn injuries with candidemia. These patients have an elevated risk for mortality according to their Acute Physiology and Chronic Health Evaluation (APACHE) II score, Charlson comorbidity index, treatment with azoles, treatment with polyenes, and duration of antifungal therapy.^16^ Among pediatric patients with burns and BSI, central-line catheters^35^ and higher *Candida* scores^17^ were each associated with increased mortality risk.

Only 3 of the included studies discussed the potential factors for poorer outcomes other than death.^24^^,^^33^^,^^34^ These articles identified only a few independent predictors. The increased risk of admission to an intensive care unit and surgical intervention among patients with burn injuries with BSIs was linked to flame or electric burns,^24^ but the risk decreased with TBSA,^34^ catecholamine use^34^ and hemofiltration.^33^^,^^34^

## Discussion

This is the first review article, to our knowledge, to comprehensively examine the incidence rates and risk factors for injury-related BSIs worldwide. Among the 48 included studies, we identified 15 studies that reported highly variable incidence rates (from 0.71 to 27.4 episodes per 1000 patient-days) (Table S4) that were largely dependent on the study context and population. Cumulative incidence was determined for all but 4 studies; the pooled cumulative incidence was 1.47% (range, 0.2-67.8). Finally, a broad range of risk factors was determined across 41 included studies that either reported risk factors for incidence or adverse outcomes from injury-related BSIs. Although some risk factors appeared important across studies, there was considerable heterogeneity regarding analyses and variable selection that led to mixed findings or, in some cases, variables considered only once and so their importance may be overinflated. Therefore, these risk factors cannot be generalized to broader population but can be applied contextually, highlighting the need for stronger study designs that capture all residents of a given area to better understand injury-related BSIs.

The designs and populations of the included studies limit the generalizability of our findings. First, most studies focused on highly selective cohorts. For example, some studies considered either adult or pediatric populations, and others focused on highly specific clinical settings or events, such as people following a particular industrial incident.^39^ Outside of the injury context, several studies were conducted to assess the overall burden of BSIs at the population level; some were conducted for specific age cohorts (eg, adult or pediatric)^58^^,^^59^ or specific types of BSI.^60^ However, there are no population-based studies of injury-related BSIs, as determined by this review. There was often a lack of detail regarding how injury populations were determined, which again directly affected the strength of the findings. More rigorously designed and reported studies are needed to strengthen our understanding of the incidence and risk factors for injuries complicated by BSIs.

The overall understanding of injury-related BSIs has important clinical implications; however, this review identified significant gaps in our knowledge. Of the 41 included studies that reported risk factors, 63% did not report adverse outcomes, and these only were reported among patients with burn injuries. As such, an understanding of the potential factors associated with attributable deaths among broader injury populations is needed. The absence of these studies affects our understanding of what interventions could help reduce poorer outcomes among injured patients with BSIs and that could ultimately save lives. Although this review provides a rigorous approach to exploring the incidence and risk factors of BSIs, there were several underrepresented groups evident in the literature. Injuries are a recognized burden in low- and middle-income countries; however, the studies in these countries were largely unaccounted for among the included studies. More studies from these poorly represented low- and middle-income countries are needed. Even though challenges are likely to exist regarding the availability of data, particularly comprehensive linked data, even single-center studies would add to the current knowledge.

This review had some limitations that may affect the interpretation of findings. The scope of evidence in this review was limited to published journal articles. Although results from articles such as case reports are not generalizable, the exclusion of these studies could limit the identification of newly identified risk factors if these were not identified among the included cohort studies. Gray literature was also not considered. Although non–English language articles were included, which is a strength because these are often underrepresented in review articles, the database searches may have missed journals that are both not indexed and only available in other languages. Although we identified a number of factors associated with risk for BSI, these do not necessarily directly imply how to change therapy for an individual patient. The present review identifies factors that could be assessed in prospective studies aimed at interventions that could reduce subsequent infection rates. Despite these limitations, this study gives a clear picture of the current knowledge of incidence rates and risk factors of injury-related BSIs.

### Conclusions

Injury-related BSI is a major complication arising as a result of injuries and can be prevented or reduced through early diagnosis, infection control measures, and timely interventions. However, the true burden of injury-related BSIs across the world is unknown, due to the lack of population-based studies on injury-related BSIs. Given the large impact of injuries in low-income countries, we recommend additional studies in these nations, whereas high-income countries need to conduct population-based studies. Because the potential factors extracted from the included studies were from different study contexts, this collection of factors summarizes most of the plausible factors from different populations and settings that can affect the development and poorer outcomes from injury-related BSIs.

## Supplementary Material

Web_Material_mxaf015
