# Correction to: Global incidence and risk factors for injury-related bloodstream infections: a scoping review

**DOI:** 10.1093/epirev/mxag014

**Published:** 2026-10-17

This is a correction to: Binuri Perera, Ashleigh Earnshaw, Kevin Laupland, Samantha Borg, Kirsten Vallmuur, Felicity Edwards, Susanna Cramb, Global incidence and risk factors for injury-related bloodstream infections: a scoping review, *Epidemiologic Reviews*, Volume 47, Issue 1, 2025, mxaf015, https://doi.org/10.1093/epirev/mxaf015.

In the originally published version of this manuscript, the supplementary material file was omitted.

The omitted document has now been added to the article.

